# Large increases in carbon burial in northern lakes during the Anthropocene

**DOI:** 10.1038/ncomms10016

**Published:** 2015-11-26

**Authors:** Adam J. Heathcote, N. John Anderson, Yves T. Prairie, Daniel R. Engstrom, Paul A. del Giorgio

**Affiliations:** 1 Groupe de Recherche Interuniversitaire en Limnologie, Département des Sciences Biologiques, Université du Québec à Montréal, Case postale 8888, Succursale Centre-ville, Montréal, Québec, Canada H3C 3P8; 2 St Croix Watershed Research Station, Science Museum of Minnesota, 16910 152nd Street North, Marine on St Croix, Minnesota 55047, USA; 3 Department of Geography, Loughborough University, Loughborough, Leicestershire LE11 3TU, UK

## Abstract

Northern forests are important ecosystems for carbon (C) cycling and lakes within them process and bury large amounts of organic-C. Current burial estimates are poorly constrained and may discount other shifts in organic-C burial driven by global change. Here we analyse a suite of northern lakes to determine trends in organic-C burial throughout the Anthropocene. We found burial rates increased significantly over the last century and are up to five times greater than previous estimates. Despite a correlation with temperature, warming alone did not explain the increase in burial, suggesting the importance of other drivers including atmospherically deposited reactive nitrogen. Upscaling mean lake burial rates for each time period to global northern forests yields up to 4.5 Pg C accumulated in the last 100 years—20% of the total burial over the Holocene. Our results indicate that lakes will become increasingly important for C burial under future global change scenarios.

## Abstract (web summary)

The rate at which carbon burial has changed in lakes in response to human-induced global change is poorly understood. Here, the authors show that carbon burial has increased significantly in remote northern lakes along with increased nitrogen deposition and warming over the last century.

## Graphical abstract

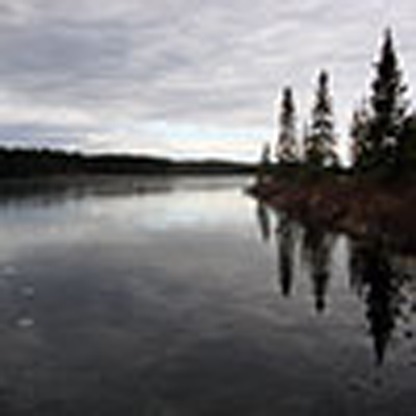

The boreal and north-temperate forests contain the highest density of freshwater lakes globally^1^ and lake sediments represent one of the largest terrestrial stocks of organic carbon (C)^2^,^3^. These lakes (hereafter referred to as northern lakes) represent an important link in the global C cycle, sequestering as much as 15% (ref. 4) and evading up to 48% of the terrestrial C that would otherwise be exported to the oceans^5^. C burial in lakes is the net balance of autotrophic fixation within the lake, transfer of C from terrestrial ecosystems, mineralization and downstream export^6^. The balance between net C source and sink in northern lakes is delicate and sensitive to global forcing^7^,^8^, thus understanding the rates at which lakes process and store C, and how that has changed over time, is critical to assessing the role of lakes in the contemporary global C cycle. As the catchments of most northern lakes are minimally disturbed by human land-use change—otherwise an important driver of burial rates^9^,^10^,^11^,^12^—organic-C burial in these systems is often calculated as an average since the last de-glaciation (that is, the Holocene epoch *ca*. 11,700 BP)^2^,^13^. There is growing evidence, however, that remote aquatic ecosystems have been affected by human-induced global change drivers^14^,^15^ since the turn of the nineteenth century, a time period dubbed the Anthropocene^16^. This raises the question as to whether the rates of organic-C processing in northern lakes can be considered static over this period and to what extent they may be sensitive to global drivers affecting climate^17^ and nutrient loading^15^,^18^ over the last 150 years.

Previous studies of organic-C burial in northern lakes have focused on either a small number of sites with a relatively high temporal resolution using ^210^Pb dating^6^,^19^ or a larger set of lakes with low temporal resolution (averaging entire Holocene organic-C stock)^2^. Few, if any, studies have examined northern lakes at high spatial and temporal resolution using consistent methodology for both radioisotopic dating and whole-basin upscaling. Here we incorporate ^210^Pb-dated sediment cores with measurements of organic carbon content from 101 lakes ranging longitudinally from Alaska to Newfoundland and spanning a latitudinal gradient from 44 to 58° N (Fig. 1a). The suite of lakes are all located within protected regions and have been relatively unimpacted by direct human disturbance to their catchment. Importantly, all rates of organic-C burial reported here have been corrected for sediment focusing and incomplete mineralization of organic C in the most recent surficial sediments (see Methods for a detailed explanation).

For this study we report significant increases in organic-C burial in relatively undisturbed northern lakes over the last 100 years. Although there is a correlation with temperature across our large spatial gradient, warming alone does not fully explain our results. Reactive nitrogen (Nr) deposition is proposed as a driver of increased organic-C burial based on concurrent data from the Greenland Ice Sheet^20^. These results are the first to show systematic increases in organic-C burial in remote northern lakes and provide further direct evidence of significant ongoing changes to biogeochemical processes in northern landscapes.

## Results

### Changes to organic-C burial rates over the Anthropocene

**Figure 1:**
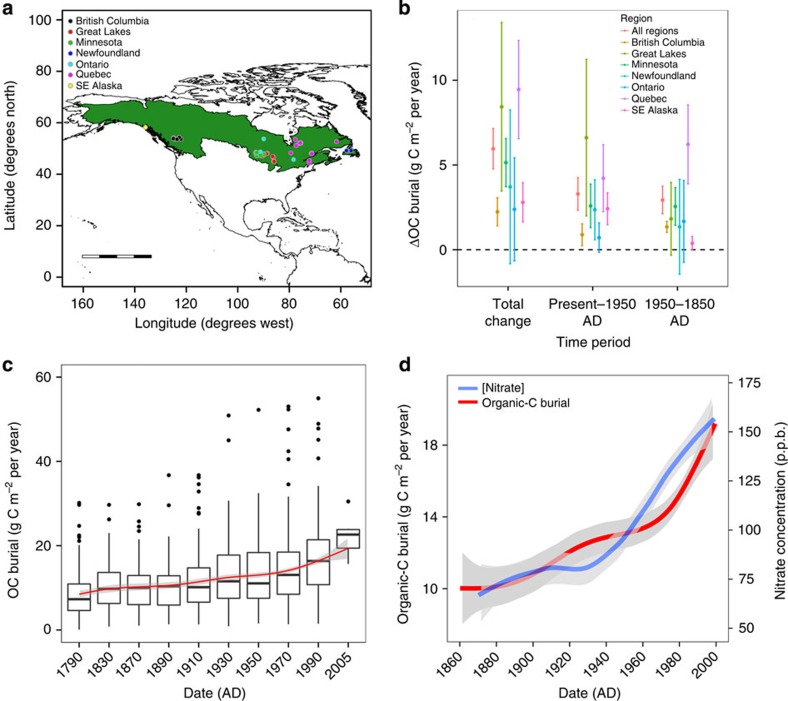
Organic-C burial in northern lakes. (**a**) Map of North America with lakes included in this study coloured by region. The boreal and northern temperate forest biome (referred to as northern forest in this study) is shaded in green. Scale bar, 2,000 km. (**b**) Mean change in organic-C (ΔOC) burial rates between time periods for lakes in this study for all regions (*n*=101), British Columbia (*n*=10), the Great Lakes Region (*n*=13), Minnesota (*n*=39), Newfoundland (*n*=4), Ontario (*n*=3), Québec (*n*=28) and southeast Alaska (*n*=4). Error bars represent the 95% confidence interval around the mean and those that do not overlap zero represent a significant positive increase (*t*-test, *P*<0.05). (**c**) OC burial versus time, binned by 20-year intervals, from 1840 to 2000 AD. All dated sections older than 1840 and younger than 2000 AD were combined into two bins due to a low density of points for those time periods. The median for all bins was fit with a LOWESS smoother (red line), to show the general trend in organic-C burial over time. Boxes represent the interquartile range (IQR) with the median shown as a black bar. Whiskers represent the minimum/maximum value within × 1.5 the IQR with points outside plotted as black dots. (**d**) LOWESS smoother for OC burial from all lakes in this study versus time (red line) and atmospherically deposited nitrate concentrations (teal line) measured from the Greenland Ice Sheet. Greenland ice core data modified from Hastings *et al.*^20^.

We estimate an average post 1900 organic-C burial rate of 13.9±0.6 g C m^−2^ per year across the northern lakes of North America—a mean rate that is three to five times higher than previously estimated Holocene averages of 2.7 C m^−2^ per year for Finland^2^ and 3.8 C m^−2^ per year for Québec^13^. Organic-C burial rates were compared between three distinct time periods: pre-1900 (before widespread industrialization in North America), 1900–1950 (the initial phase of population and industrial growth), and 1950 to present (the ‘Great Acceleration' in global industrialization following World War II^16^). There were significant (*P*<0.001) increases in organic-C burial from pre-1900–1950 and 1950 to present, although the magnitude of the mean difference between time periods varied by region (Fig. 1b,c). With all lakes combined, organic-C burial increased from an average of 9.5±0.5 g C m^−2^ per year before 1900 to 12.3±0.8 g C m^−2^ per year between 1900 and 1950, and to 15.0±0.8 g C m^−2^ per year between 1950 and present (Table 1). This amounts to a mean lake-specific increase of 5.5 g C m^−2^ per year across all lakes since 1900.

### Sensitivity of boreal lakes to climate

**Figure 2:**
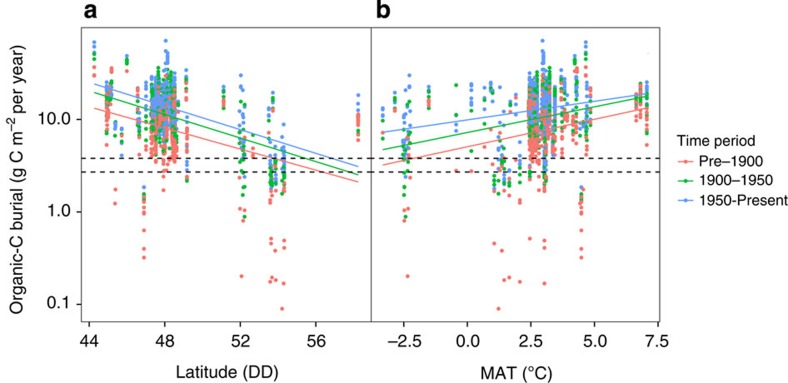
Geographic drivers of organic-C burial. Organic-C burial rates for 101 northern lakes over a latitudinal (**a**) and temperature (**b**) gradient. The colour of the points represents three different time periods: before 1900 AD (orange), 1900–1950 AD (green) and 1950 to present day (blue). Solid lines represent least square regressions for each time period and dashed lines represent previously estimated Holocene burial rates for Finland^2^ (below; 2.7 g C m^−2^ per year) and Québec^13^ (above; 3.8 g C m^−2^ per year).

There are several potential human-induced drivers that could have impacted the rates of carbon burial over the last century, including observed increases to temperature in northern-forested biomes. Previous experimental results have suggested increasing temperature could decrease C burial in lakes^17^, yet our data show a negative correlation with latitude, (*r*^2^_adj_=0.20, F_3,1356_=117.2, *P*<0.001; Fig. 2a), which would in turn suggest a positive relationship with temperature. We explored this relationship further by comparing organic-C burial rates to mean annual temperature (MAT) across our data set (range: −3 to 7 °C MAT) and found a significant positive correlation of organic-C burial to MAT (*r*^2^_adj_=0.13, F_3,1356_=66.22, *P*<0.001; Fig. 2b) resulting in a slope of +1 g C m^−2^ per year per 1 °C MAT across all time periods. It would thus appear that, contrary to experimental results, at the ecosystem level temperature has a positive effect on organic-C burial. This relationship may reflect a direct effect of temperature, but most probably combines effects of other drivers that are themselves linked to temperature.

The observed average increase in organic-C burial of 5.5 g C m^−2^ per year since 1900 across all the lakes requires a comparable increase in temperature to have occurred over time for warming alone, to explain changes seen in these lakes. This is not the case, however, as the mean and maximum change in MAT for regions in this study is +0.8 and 1.0 °C since 1900. This indicates that, based on our regression model, temperature can only account for a maximum of 18% of the increase seen in organic-C burial. In addition, we found no significant correlation between the estimated contemporary change in burial and the corresponding change in MATs compiled from long-term (70–100 years) regional monitoring stations for the period of 1900–1950 or 1950 to present (Supplementary Fig. 1). Regional changes in temperature cannot fully explain the observed patterns in lake organic-C burial.

## Discussion

Our revised rate of recent organic-C burial in northern lakes is still modest relative to annual anthropogenic C emissions (8 Pg C per year) and the net atmospheric C increase (2–4 Pg C per year)^21^, but it is nevertheless significant relative to other regional C components. For example, there are predictions of increasing greenhouse gas emissions from northern aquatic networks resulting from both direct temperature increases^17^ and indirectly through increases in the loading and subsequent remineralization of terrestrial organic matter to these surface waters^22^. The increase in lake organic-C burial, which will offset to some degree these shifts, needs to be considered in future scenarios of net regional C balances.

The most recent organic-C burial rates calculated here (15 g C m^−2^ per year) for northern lakes is still lower than those observed in culturally eutrophic lakes in North America and Europe (∼50–150 g C m^−2^ per year)^9^,^11^; however, this upshift will nevertheless have a large effect on calculations of organic-C buried by lakes globally because of the density of lake surfaces in northern landscapes. The boreal forest biome alone accounts for 28% of global freshwater lake area^1^,^2^, despite only covering 14% of the Earth's total land area^23^, meaning that even slight changes in rates will have a large impact at a global scale.

Lakes account for 10% to over 20% of the total area of northern forested ecosystems^2^,^23^,^24^ and estimates of lake density on the Earth's non-glaciated land surface has doubled in the last 20 years^1^,^25^,^26^. Furthermore, we continue to refine our understanding of how lakes process carbon over time and space, to improve global upscaling predictions^27^,^28^,^29^. Previous estimates of global-C processing by lakes used rates of organic-C burial in natural lakes averaged over 10,000 years^2^,^27^,^28^, whereas our results indicate a systematic elevation of burial rates over the last 100 years. This implies a significant underestimate of modern organic-C burial for northern lakes worldwide. Upscaling our post-1950 organic-C burial rates to the 2.4 million km^2^ of northern forest in North America^23^ yields an annual burial rate of 2.5–7.2 Tg C per year, 37% of which (0.9–2.5 Tg) would have been missed using average Holocene burial rates. If we further upscale this rate to a global estimate of the northern forested biome (14.2 million km^2^)^2^,^23^, the current burial rate for the ensemble of northern lakes would be on the order of 0.02–0.04 Pg C per year. This flux is of the same order of magnitude as the current estimates for organic-C burial for all the world's natural lakes combined (0.07 Pg C per year)^28^. By revising upwards previous organic-C burial derived from Holocene accumulation rates, we estimate 2.0–4.5 Pg of organic-C have been buried in sediments of northern lakes during the last 100 years, the period of greatest disruption of global biogeochemical cycles. This would account for 10 to 22% of total C stocks sequestered in northern lake sediments over the entire Holocene^2^.

Our results indicate that climate cannot fully explain this increased rate of organic-C burial observed over the Anthropocene and thus other large-scale human-induced landscape changes could be responsible^10^,^11^. Direct catchment impacts such as agriculture and human development are nearly non-existent in the catchments of the study lakes, and although logging has historically occurred in some regions it is generally less extensive compared with northern forests of Europe (for example, Scandanavia^30^). Although we cannot rule out some transfer of soil carbon into lakes as a result of logging, previous work has shown that even extensively logged catchments in this region have not shown a significant increase in carbon burial over non-forested catchments^31^. Beyond human manipulation of the landscape, there is a potential for changing fire patterns in the northern forests in response to changing climate. Whereas increasing catchment fire frequency could lead to decreased storage of organic-C in forest soils, and some loading of organic-C to lakes, shifts in fire patterns are asynchronous and variable across North America^32^.

Given the timing of increased organic-C burial and the absence of coinciding direct catchment disturbance, other processes operating at a continental or global scale must be influencing contemporary organic-C burial in northern lakes. One probable driver is the enhanced primary production of the forested catchment, stimulated by atmospheric deposition of anthropogenically derived reactive nitrogen (Nr). Nr deposition has increased by an order of magnitude globally since 1860 (ref. 18) and has been detectable via isotopic signatures in northern lake sediments and elevated nitrate concentrations in the Greenland Ice Sheet since the beginning of the twentieth century^15^,^20^. The timing of this increase in Nr deposition closely matches the rise in organic-C burial seen in these lakes (Fig. 1d), although this relationship is not entirely consistent through time.

Although such evidence is correlative, and thus does not demonstrate strictly causal links, the coherence in trends provides at least a preliminary mechanistic hypothesis. Long-term experimental studies in the boreal forest have shown that forest soil C sequestration increases and mineralization decreases in response to atmospheric Nr deposition^33^, leading to a larger pool of organic C that could be transported to lakes and these findings have been corroborated by process-based models developed to simulate forest production and soil sequestration over the last 100 years^34^. It is also possible that Nr deposition is leading to fertilization of the lakes themselves. Previous work has shown that Nr deposition may alter the nitrogen to phosphorus stoichiometry of northern lakes^35^,^36^ and distinct transitional zones among phytoplankton are evident as early as 1850 AD in arctic and alpine lakes^37^. The degree to which these changes in nutrient stoichiometry and phytoplankton composition cause or reflect increased organic-C burial in these lakes are unclear, but this provides a complementary mechanism from which Nr deposition could be driving the changes seen in this study. These conclusions are supported by the previous work of Kortelainen *et al.*^38^ who found that N and organic-C burial were strongly correlated in a randomly sampled set of boreal lakes in Finland. Such results imply that we need to consider how the carbon dynamics of aquatic systems could change under future patterns of atmospheric Nr deposition.

The trends in Nr deposition from the Greenland Ice Sheet vary over time in relation to organic-C burial in lakes, especially between 1910 and 1940 when Nr deposition appears relatively stable, while organic-C burial continues to increase. The two trends are somewhat reversed between 1930 and 1960 when Nr deposition again rises and organic-C burial levels off (Fig. 1d). This offset could indicate a time lag between Nr stimulation of terrestrial organic-C fixation and the subsequent transport of that carbon into aquatic systems. We should be careful, however, not to overinterpret divergence between Nr deposition derived from a single ice core and our data set of organic-C burial averaged over many lakes. Trends derived from a single site, such as the Greenland ice core, are likely to incorporate more stochastic variability that is smoothed out across our larger data set of the northern lakes.

In addition to changes in organic-C burial fuelled by Nr deposition over the last century, increasing export of dissolved organic carbon (DOC) from catchments has led to the observations of significant browning in northern lakes over the past 30 years^39^,^40^. These changes in DOC export to lakes have been attributed to increasing hydrologic runoff^4^ and reversal of landscape acidification in unbuffered systems following a reduction in sulphate deposition^40^. As this additional DOC may be sequestered in sediments by flocculation^28^, lakes in regions sensitive to brownification, such as the eastern Canadian Shield, may see even larger increases to organic-C burial.

Beyond the magnitude and regional significance of this contemporary organic-C burial in lakes, the recent shifts are probably significant from a functional landscape perspective. Lake organic-C burial is complex and necessarily integrates a number of key aquatic and terrestrial processes related to the production, transport and processing of organic C. It is clear from our data and results of other recent studies^41^ that temperature plays an important role in the spatial patterns of organic-C burial in lakes. Our data have shown, however, that a temporal increase in organic-C burial even greater than can be explained by warming has occurred over the last century. We have proposed atmospheric Nr deposition as one potential driver through the stimulation of forest soil C sequestration and lateral transfer of DOC through hydrologic pathways. Although the link between N fertilization and C sequestration has been intensively studied in the terrestrial literature^33^,^34^, we know relatively little about how this linkage is transferred to aquatic systems^38^. Further study is needed to corroborate our findings or propose alternative mechanisms, but it is clear that lakes are acting as sentinels and integrators of regional processes, and that these large-scale changes in lake organic-C burial are signalling major shifts in the overall biogeochemical functioning of northern landscapes.

## Methods

### Measuring organic-C burial from lake sediments

Organic-C burial rates in this study are a combination of newly collected and previously published data (see Supplementary Table 1 for a list of sources and citations) from northern forested lakes with minimal direct catchment disturbance. Sample size was limited to lakes sampled by the authors and previously published work where reliably dated sediment core records were available. Organic-C burial was estimated from sediment cores collected from the deep central basin of each lake^42^ and sectioned at 0.5- to 1-cm intervals. The percentage of organic C in each section was determined by loss on ignition^43^. Dry mass accumulation rates were calculated based on ^210^Pb, a naturally occurring radioisotope with a half-life of 22.3 years, following the constant rate of supply model^44^.

There are two factors that need addressing when using lake sediments to determine organic-C burial rates: (1) post-deposition mineralization and (2) sediment focusing. We have considered both of these issues in our methodological approach. First, previous work with repeated sediment coring has shown that over a period of 27 years, 87% of sedimentary C-loss happened within the first 5 years and older sediments lost <1% of sedimentary C per year^45^. Based on modelled decomposition rates in a boreal lake taken from the literature (see Supplementary Fig. 2), we excluded sediment younger than 10 years entirely from our analysis, to focus on time periods where post-depositional mineralization no longer accounts for a significant loss in C relative to organic-C burial rates. Second, as sediments are unevenly deposited across a lake bottom due to post-depositional horizontal transport, referred to as sediment focusing, site-specific burial rates for organic-C were corrected to a whole-basin rate using a focusing factor based on the observed versus expected atmospheric flux of ^210^Pb to the sediment^9^,^10^. Where measured atmospheric fluxes of ^210^Pb were not available, we assumed an average ^210^Pb flux of 100 Bq kg^−1^ per year per 1,000 mm of precipitation^46^, with annual precipitation derived from the world climate data interpolation at 1-km spatial resolution^47^ (WORLDCLIM; www.worldclim.org). Mean annual temperature for each site was also taken from the WORLDCLIM database at the same resolution. For site-specific observed temperature change, the closest weather station with long-term (50 to 100+ years) data available was selected from the Global Historical Climatology Network, version 3 (www.ncdc.noaa.gov/ghcnm/v3.php). From these data, temporal changes in MAT for the various lake regions were modelled using a locally weighted, polynomial-regression (LOWESS) smoother^48^. Changes in MAT (ΔMAT) were calculated as the difference in MAT averaged over the same time periods as carbon burial (see below). Long-term trends in MAT for each regional subset are shown in Supplementary Fig. 3.

### Data treatment

Organic-C burial rates were separated into three time periods based on global anthropogenic activity before and after the beginning of the Anthropocene: Pre-1900, 1900–1950, 1950–2000 (ref. 16). Pre-1900 was considered background or very low disturbance in North America, as well as limited global atmospheric emissions. The period 1900–1950 was considered a transitory period with increasing human population and industrialization. The period 1950 to present was considered the modern period and reflective of the post-World War II increase in both the human population and industrialization. Statistical comparisons for significant increases between periods were made using one-sided paired *t*-tests for a difference in organic-C burial greater than zero.

## Additional information

**How to cite this article:** Heathcote, A. J. *et al.* Large increases in carbon burial in northern lakes during the Anthropocene. *Nat. Commun.* 6:10016 doi: 10.1038/ncomms10016 (2015).

## Supplementary Material

Supplementary Information Supplementary Figures 1-3, Supplementary Table 1 and Supplementary References.

## Figures and Tables

**Table 1:** Regional and combined averages (and s.d.) for organic-C burial before and following the Anthropocene.

**Region**	**Pre-1900 OC burial (g** **m**^**−2**^ **per year)**	**1900–1950 OC burial (g** **m**^**−2**^ **per year)**	**1950-Present OC burial (g** **m**^**−2**^ **per year)**
British Columbia (CA)	1.2 (0.9)	2.5 (0.5)	3.5 (1.3)
Great Lakes (USA)	10.8 (7.3)	13.9 (7.2)	19.8 (13.4)
Minnesota (USA)	9.8 (4.3)	13.4 (6.4)	15.4 (5.7)
Newfoundland (CA)	8.4 (7.1)	11.9 (10.7)	15.7 (12.3)
Ontario (CA)	11.0 (2.9)	12.6 (4.5)	11.4 (5.3)
Quebec (CA)	10.2 (10.3)	11.5 (11.6)	16.7 (13.9)
SE Alaska (USA)	9.8 (1.6)	9.7 (1.6)	12.5 (3.1)
All Regions	9.5 (5.8)	12.3 (7.6)	15.0 (9.4)

C, carbon.
