# Supplementary material for: Large increases in carbon burial in northern lakes during the Anthropocene
Source: Nat Commun. 2015 Nov 26;6:10016. doi: 10.1038/ncomms10016 (PMC4674672; doi:10.1038/ncomms10016)
Supplement: Supplementary Information — Supplementary Figures 1-3, Supplementary Table 1 and Supplementary References. [file ncomms10016-s1.pdf]

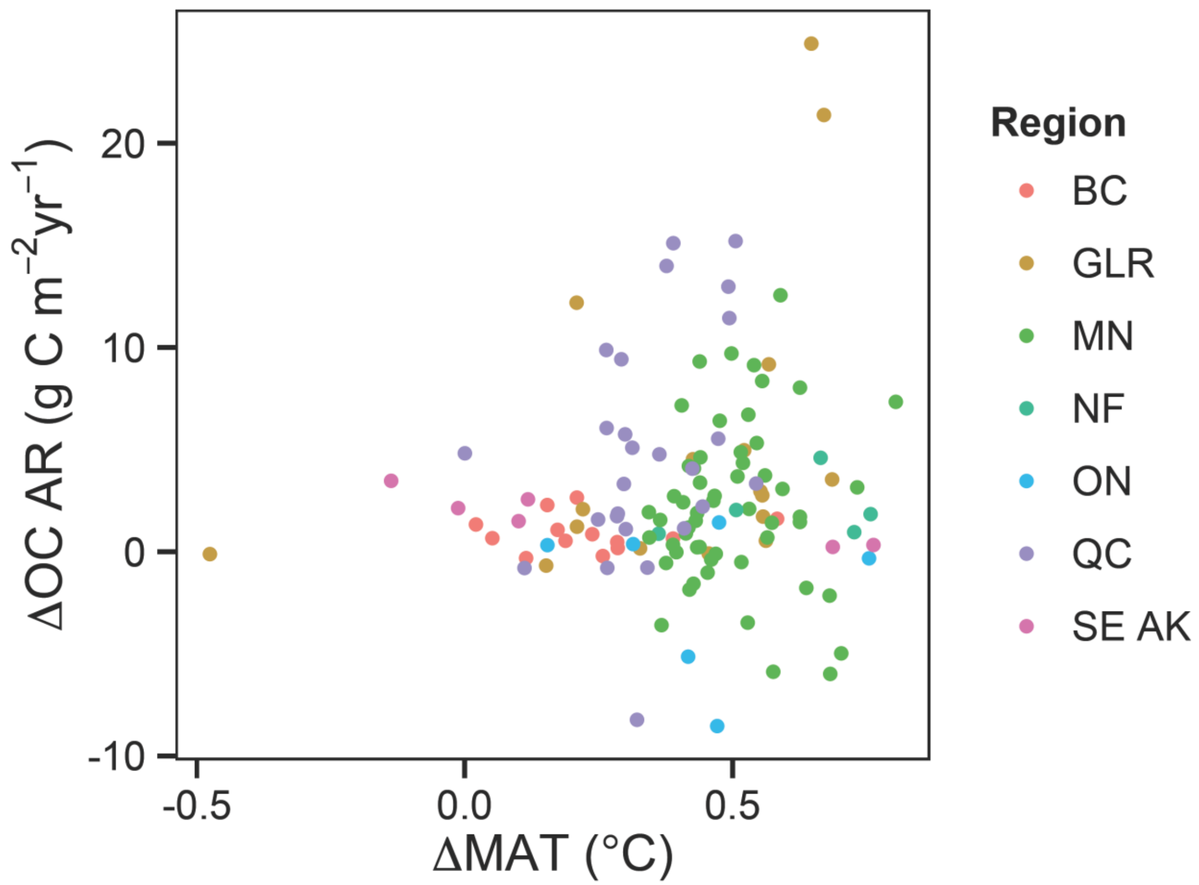

Supplementary Figure 1. Change in organic-C burial (ΔOC AR) vs change in observed surface mean annual temperature (ΔMAT) for lakes in this study. Only lakes where surface temperature data were recorded within 100 km for > 100 years (n = 100) are shown. The regions are British Columbia (BC), Great Lakes Region (GLR), Minnesota (MN), Newfoundland (NF), Ontario (ON), Québec (QC), and southeast Alaska (SE AK).

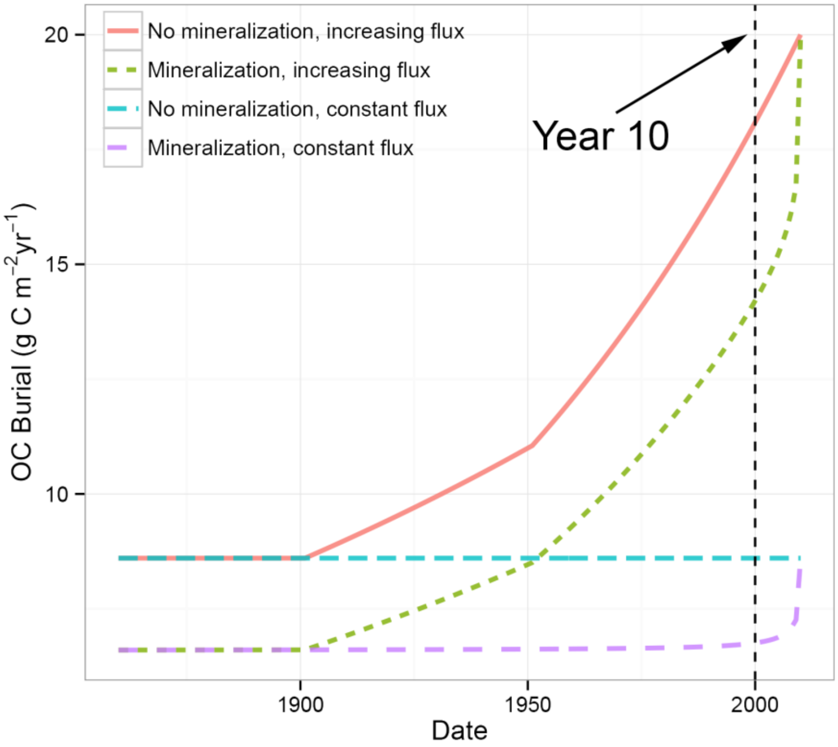

Supplementary Figure 2. Simulation of organic-C burial and mineralization over time under four scenarios. Orange and green lines represent constant burial prior to 1900, a 0.5% increase per year from 1900-1950, and a 1% increase per year from 1950 to present. Mineralization is modelled in the green line and ignored in the orange line. Blue and purple lines represent constant burial rates over time, with mineralization modelled for the purple line and ignored in the blue line. Dashed vertical line represents sediment that is older than 10 years, the cut off used in this study, and marks the transition from a slope primarily driven by mineralization, to one primarily driven by changing rates of organic-C burial. The numbers used for organic-C burial in the simulation are hypothetical, but the overall magnitude and rate of change to burial rates are similar to the average seen in northern lakes for this study. All mineralization rates based on observations from repeated sediment cores conducted by Gälman et al.[1].

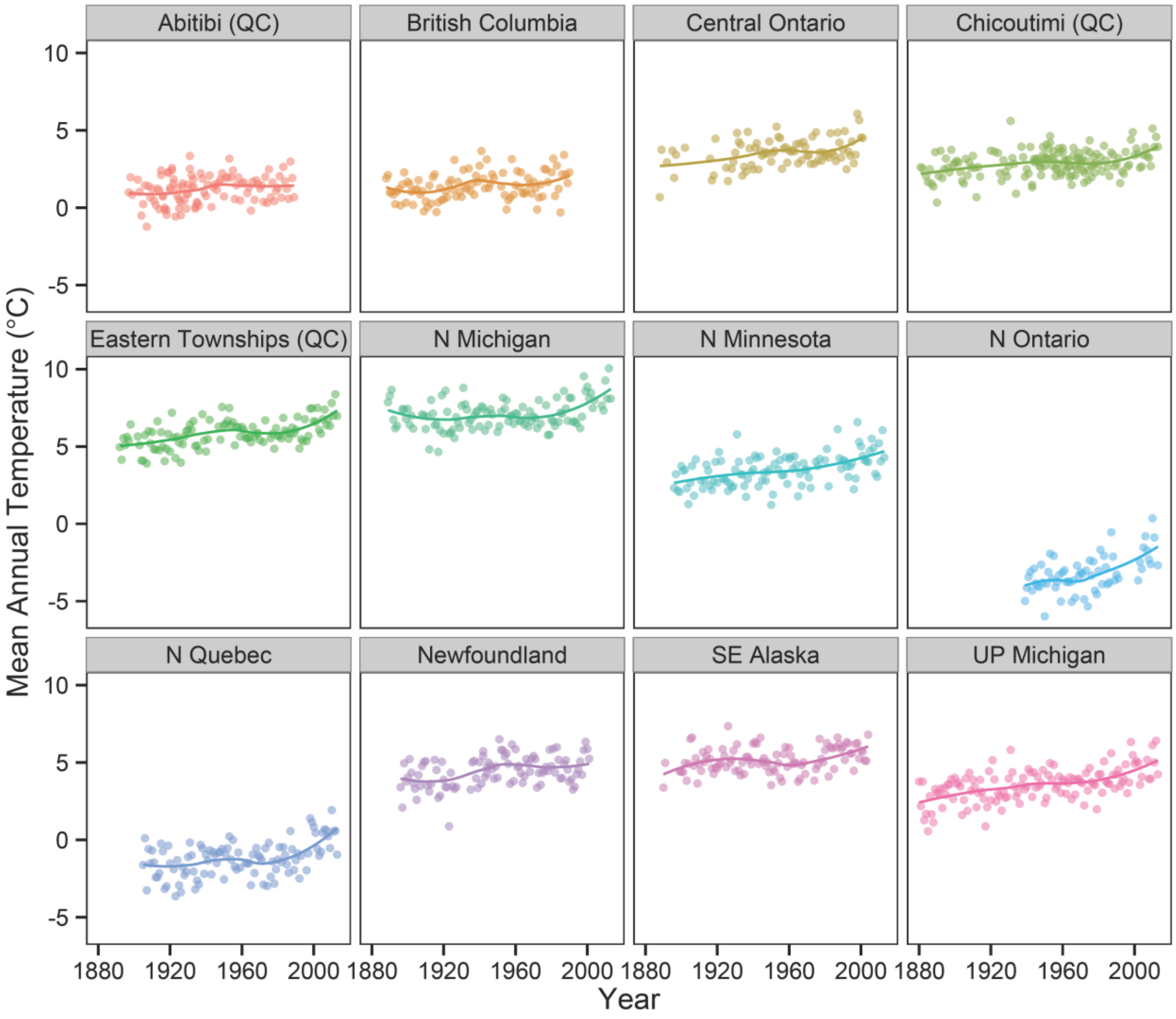

Supplementary Figure 3. Mean annual temperature over time for climate stations near the lakes from this study. Most records reflect continuous measurements at a single station, with the exception of Abitibi, Chicoutimi, and Newfoundland, where data from two nearby stations were combined to form a complete record over time. N = north, SE = southeast, QC = Québec, and UP = upper peninsula of Michigan.

Supplementary Table 1. Location and literature source for the lakes included in this study.

Lake	Region	Latitude (N)	Longitude (W)	Citation
Boomerang	British Columbia	53.6766	124.5165	2
Jakes	British Columbia	54.3245	122.7116	2
Justine	British Columbia	54.2118	124.9375	2
Laurie	British Columbia	53.8783	124.8881	2
Pitoney	British Columbia	53.6303	122.0334	2
Secord	British Columbia	53.6284	124.3368	2
Tang	British Columbia	54.3231	122.7846	2
Unnamed	British Columbia	53.8473	125.0168	2
Upper Summit	British Columbia	54.2945	122.7174	2
Woodcock	British Columbia	53.5824	123.5961	2
Ahmik	Great Lakes	48.1479	88.5415	This study
Bass	Great Lakes	44.9224	85.8832	This study
Beaver	Great Lakes	46.5652	86.3436	This study
Florence	Great Lakes	45.0109	86.1199	This study
Grand Sable	Great Lakes	46.6479	86.0336	This study
Harvey	Great Lakes	48.0507	88.7960	This study
Lake Superior	Great Lakes	46.8982	86.5007	3
Manitou	Great Lakes	45.1269	86.0237	This study
Moskey Bay (Superior)	Great Lakes	48.0690	88.6357	This study
Outer	Great Lakes	47.0075	90.4597	This study
Richie	Great Lakes	48.0409	88.7024	This study
Shell	Great Lakes	44.9474	85.8994	This study
Siskwit	Great Lakes	47.9992	88.8001	3
Agnes	Minnesota	47.5280	92.8116	4
Alton	Minnesota	47.8631	90.9083	4
Astrid	Minnesota	48.1107	92.3289	4
August	Minnesota	47.7611	91.6064	4
Bean-Lake	Minnesota	47.3089	91.3007	4
Bear	Minnesota	47.2847	91.3443	4
Brule	Minnesota	47.9325	90.6721	4
Cruiser	Minnesota	48.4986	92.8063	4
Dunnigan	Minnesota	47.7074	91.6323	4
Dyers	Minnesota	47.5286	90.9807	4
Ek	Minnesota	47.5286	92.8352	4
Grassy	Minnesota	47.8035	92.0432	4
Greenstone	Minnesota	47.9324	91.6056	4
Kabetogama	Minnesota	48.4583	93.0419	4
Kjostad	Minnesota	48.1101	92.6113	4

Lac La Croix	Minnesota	48.3497	92.1341	4
Little	Minnesota	48.3334	93.0964	4
Little Trout	Minnesota	48.3972	92.5227	4
Little Wilson	Minnesota	47.6581	91.0666	4
Locator	Minnesota	48.5412	93.0056	4
Loiten	Minnesota	48.5256	92.9233	4
Maude	Minnesota	48.1092	92.3529	4
Mukooda	Minnesota	48.3337	92.4903	4
Namakan	Minnesota	48.4353	92.5836	4
Ninemile	Minnesota	47.5794	91.0833	4
Nipisiquit	Minnesota	47.3556	91.2474	4
Peary	Minnesota	48.5261	92.7708	4
Rainy	Minnesota	48.6244	93.0117	4
Ryan	Minnesota	48.5193	92.7074	4
Sawbill	Minnesota	47.5280	90.8802	4
Shoepack	Minnesota	48.5031	92.8802	4
Speckled Trout	Minnesota	47.5105	89.8451	4
Swamp	Minnesota	47.9513	89.8581	4
Tettegouche	Minnesota	47.3456	91.2685	4
Tooth	Minnesota	48.3983	92.6430	4
Wilson	Minnesota	47.6750	91.0836	4
Windy	Minnesota	47.7289	91.0766	4
Wolf (Johnson)	Minnesota	47.3756	91.1893	4
Clever	Newfoundland	49.1700	57.7680	5
Frank	Newfoundland	49.1780	57.6480	5
Tomtit	Newfoundland	49.1720	57.7900	5
Topsail	Newfoundland	49.1330	56.0130	5
Big Trout	Ontario	53.7213	89.9426	3
Eva	Ontario	48.7132	91.1859	3
Opeongo	Ontario	45.7180	78.3608	3
Bousquet	Quebec	48.0286	71.4707	This study
Brendan	Quebec	52.0653	75.5028	6
Cantin	Quebec	47.9761	71.1566	This study
Clarence-Gagnon	Quebec	48.0915	71.5292	This study
Clarkie	Quebec	52.2278	75.4894	6
EM-320	Quebec	52.1656	76.1208	6
Faniant	Quebec	48.1268	71.2642	This study
Fraser	Quebec	45.3881	72.1780	This study
Grand Lac Montagnais	Quebec	47.9186	71.2057	This study
Labyrinthe	Quebec	52.2261	75.7136	6
Lac 11	Quebec	52.1525	75.7600	6
Lac 2	Quebec	52.1322	75.8192	6
Lac 34	Quebec	51.9847	75.7669	6

Lac 40	Quebec	52.0294	75.5236	6
Lac 60	Quebec	52.2317	75.7617	6
Lac 66	Quebec	51.9600	76.0097	6
Lac 8	Quebec	52.1319	75.7239	6
Dasserat	Quebec	48.2492	79.4063	3
Richelieu	Quebec	48.1560	71.3509	This study
Levi Pond	Quebec (VT, USA)	44.2671	72.2275	3
Minipi	Quebec (LB, CA)	52.6679	61.7243	3
Mistumis	Quebec	52.1617	76.1808	6
Natel	Quebec	52.1847	75.7119	6
Q27	Quebec	53.5072	77.6986	3
Q6	Quebec	51.1156	77.3262	3
Simoncouche	Quebec	48.2400	71.2533	This study
Stukely	Quebec	45.3638	72.2531	This study
Tourangeau	Quebec	47.9298	71.2432	This study
Cliff	SE Alaska	58.2400	135.8420	5
Goldeneye	SE Alaska	58.2470	135.8330	5
Rectangle	SE Alaska	58.2370	135.8770	5
Sapsucker	SE Alaska	58.2330	135.8230	5

Supplementary References

1. Gälman, V., Rydberg, J., Sjöstedt de-Luna, S., Bindler, R. & Renberg, I. Carbon and nitrogen loss rates during aging of lake sediment: Changes over 27 years studied in varved lake sediment. *Limnol. Oceanogr.* **53,** 1076–1082 (2008).

2. Laird, K. & Cumming, B. A regional paleolimnological assessment of the impact of clear-cutting on lakes from the central interior of British Columbia. *Can. J. Fish. Aquat. Sci.* **58,** 492–505 (2001).

3. Muir, D. C. G. *et al.* Spatial trends and historical inputs of mercury and lead in eastern and northern Canada inferred from lake sediment cores. *Environ. Sci. Technol.* **43,** 4802–4809 (2009).

4. Anderson, N. J., Dietz, R. D. & Engstrom, D. R. Land-use change, not climate, controls organic carbon burial in lakes. *Proc. R. Soc. B Biol. Sci.* **280,** 20131278 (2013).

5. Engstrom, D. R. *et al.* Atmospheric Hg emissions from preindustrial gold and silver extraction in the Americas: A reevaluation from lake-sediment archives. *Environ. Sci. Technol.* **48,** 6533–6543 (2014).

6. Ferland, M.-E., del Giorgio, P. A., Teodoru, C. R. & Prairie, Y. T. Long-term C accumulation and total C stocks in boreal lakes in northern Québec. *Global Biogeochem. Cycles* **26,** GB0E04 (2012).
